# Effects of Livergol and Hydroalcoholic Extracts of Barberry, Jujube, and Flixweed on Oxidative Stress and Inflammatory Gene Expression in a MASLD Animal Model

**DOI:** 10.1155/mi/9913600

**Published:** 2026-06-30

**Authors:** Elham Bahreini, Farzad Sadri, Mohammad Babaei, Yaser Mohammadi, Zahra Hemmati, Taraneh Rezaei

**Affiliations:** ^1^ Department of Biochemistry, School of Medicine, Iran University of Medical Sciences, Tehran, Iran, iums.ac.ir; ^2^ Geriatric Health Research Center, Birjand University of Medical Sciences, Birjand, Iran, bums.ac.ir; ^3^ Department of Basic Sciences, Faculty of Veterinary Medicine, Bu-Ali Sina University, Hamedan, Iran, basu.ac.ir; ^4^ Student Research Committee, Iran University of Medical Sciences, Tehran, Iran, iums.ac.ir

**Keywords:** *Berberis vulgaris*, *Descurainia sophia*, livergol, metabolic dysfunction-associated steatotic liver disease (MASLD), *Ziziphus jujuba* Mill

## Abstract

**Background:**

Metabolic dysfunction‐associated steatotic liver disease (MASLD) is known as one of the most common metabolic disorders. The present study aimed to investigate the effects of livergol and hydroalcoholic extracts of barberry, jujube, and flixweed in an animal model of MASLD.

**Methods:**

Forty‐eight male Wistar rats were randomly assigned to six groups (*n* = 8): (1) Normal control, (2) MASLD, (3) Barberry (100 mg/kg), (4) Jujube (400 mg/kg), (5) Flixweed (100 mg/kg), and (6) Livergol (200 mg/kg). MASLD was induced using a high‐fat diet (HFD). Animals received 70% hydroethanolic extracts from the plants investigated orally for 8 weeks. At the end of the intervention, serum and liver tissue samples were collected.

**Results:**

The results showed that treatment with barberry, jujube, and livergol significantly improved body weight control and lipid profile parameters. Barberry showed the strongest lipid‐lowering effect, reducing triglycerides (TG) and low‐density lipoprotein (LDL)‐C by ~32.0% and ~27.3%, respectively. Jujube produced the greatest reduction in malondialdehyde (MDA) levels (~25.8%), whereas barberry most effectively increased total antioxidant capacity (TAC) (~12.5%) and thiol levels (~8.7%). Barberry, jujube, and livergol also significantly downregulated proinflammatory cytokine genes (interleukin‐1 beta [IL‐1β], tumor necrosis factor‐alpha [TNF‐α], and interleukin‐6 [IL‐6]) and increased interleukin‐10 (IL‐10) expression. Additionally, histopathological indices of liver tissue improved in the treated groups. Flixweed showed only limited and mostly nonsignificant effects.

**Conclusion:**

Livergol and the hydroalcoholic extracts of barberry and jujube exerted significant hepatoprotective, antioxidant, and anti‐inflammatory effects against MASLD‐induced liver injury, with barberry demonstrating the most prominent therapeutic efficacy. These findings suggest that barberry may represent a promising candidate for future translational and clinical investigations in MASLD management.

## 1. Introduction

Metabolic dysfunction‐associated steatotic liver disease (MASLD) is the recently adopted terminology for steatotic liver disorders occurring in the context of metabolic dysfunction. The new nomenclature replaces the former term nonalcoholic fatty liver disease (NAFLD) and reflects the current understanding of the metabolic drivers underlying disease development [1]. The economic burden of this disease is estimated to exceed 103 billion dollars annually in the United States [2]. The pathophysiology of NAFLD involves multiple complex and interconnected mechanisms, including hepatic lipid accumulation, oxidative stress, mitochondrial dysfunction, and inflammatory responses [3]. Persistent hepatic inflammation is considered a key event in the transition from simple steatosis to MASH (metabolic dysfunction‐associated steatohepatitis) and contributes substantially to subsequent fibrogenesis and disease progression [4]. In this process, activated Kupffer cells and damaged hepatocytes lead to the production and secretion of several inflammatory cytokines, including tumor necrosis factor‐alpha (TNF‐α), interleukin‐6 (IL‐6), and interleukin‐1 beta (IL‐1β) [5]. These inflammatory mediators contribute to disease progression through activation of nuclear factor kappa B (NF‐κB) and mitogen‐activated protein kinase (MAPK) signaling pathways [6]. In addition, the anti‐inflammatory cytokine interleukin‐10 (IL‐10) plays an important regulatory role in modulating hepatic inflammation, and reduced IL‐10 expression has been reported to be associated with NASH severity [5, 6].

Despite significant advances in understanding the molecular mechanisms of MASLD, no drug has yet been specifically approved by the US Food and Drug Administration (FDA) for this disease, and current therapeutic approaches are mainly focused on lifestyle modification, weight reduction, and control of metabolic risk factors [7]. In recent years, herbal medicine has emerged as a promising therapeutic strategy for MASLD management [8, 9]. The selection of plants investigated in this study was based on their traditional use in metabolic disorders, documented bioactive composition, and emerging scientific evidence supporting their therapeutic potential in liver diseases.

Jujube (*Ziziphus jujuba* Mill.), widely cultivated in Asia and the Middle East, has been used for centuries in traditional medicine systems to treat gastrointestinal and metabolic disorders [10, 11]. This fruit is rich in bioactive compounds, including cyclopeptide alkaloids (jujubosides A and B), triterpenic acids (betulinic acid and oleanolic acid), and diverse polyphenolic compounds such as catechin, epicatechin, and rutin [12]. Despite studies conducted to date, the precise effects of jujube extract on inflammatory cytokine profiles in MASLD—particularly regarding modulation of IL‐1β and IL‐10—remain incompletely understood.

Barberry (*Berberis vulgaris* L.), a plant native to Iran, contains the isoquinoline alkaloid berberine as its main bioactive compound, along with other alkaloids such as palmatine and berbamine, as well as flavonoids and phenolic acids [8]. Berberine has attracted considerable scientific attention due to its pleiotropic pharmacological properties, including lipid‐lowering, anti‐inflammatory, and insulin‐sensitizing effects [13, 14]. Notably, studies focusing specifically on barberry extract remain limited.

*Descurainia sophia* Webb ex Prantl, also known as flixweed, is a member of the Brassicaceae family and has traditionally been used in Iranian and Chinese medicine for the treatment of gastrointestinal and inflammatory conditions [15]. This plant is distinguished by its high content of mucilaginous polysaccharides, soluble dietary fibers, and glucosinolates, along with flavonoid glycosides such as isorhamnetin and quercetin derivatives [9]. Emerging evidence suggests that *Descurainia sophia* polysaccharides exert prebiotic effects by modulating gut microbiota composition and increasing the production of short‐chain fatty acids (SCFAs), which play critical roles in maintaining metabolic homeostasis and regulating the gut–liver axis [9, 15]. However, direct evidence regarding its effects on hepatic inflammatory gene expression in MASLD models is limited, representing an important knowledge gap.

Milk thistle (*Silybum marianum* L.), marketed in some regions under the trade name Livergol, is one of the most extensively studied hepatoprotective plants worldwide. Its principal active component, silymarin, is a complex of flavonolignans, including silybin, silydianin, and silychristin. Silymarin exhibits strong antioxidant properties through free‐radical scavenging, increasing intracellular glutathione levels, and inhibiting lipid peroxidation [16]. Notably, most clinical studies have focused on the normalization of liver enzymes and metabolic parameters, while comprehensive evaluations of inflammatory cytokine modulation at the molecular level remain scarce [17, 18].

To further strengthen the phytochemical rationale underlying these interventions, the major bioactive constituents previously identified in barberry, jujube, flixweed, and milk thistle (the principal component of livergol) are summarized in Table 1. Despite the individual therapeutic potential of these plants, several critical knowledge gaps remain. First, although purified bioactive compounds from these plants have been studied, the efficacy of their hydroalcoholic extracts has not been systematically compared. Second, the specific effects of these extracts on hepatic inflammatory gene expression profiles—particularly the balance between proinflammatory cytokines (TNF‐α, IL‐1β, and IL‐6) and the anti‐inflammatory cytokine IL‐10—have not been comprehensively evaluated in a controlled experimental setting. Third, the comparative effectiveness of these plants in modulating inflammation‐driven MASLD progression remains unclear, limiting evidence‐based selection for therapeutic applications. Therefore, the present study was designed to address these gaps by investigating and comparing the effects of livergol and hydroalcoholic extracts of jujube, barberry, and flixweed on the expression levels of key hepatic inflammatory genes (TNF‐α, IL‐1β, IL‐6, and IL‐10), oxidative stress markers (total antioxidant capacity [TAC], malondialdehyde [MDA], and thiol groups), biochemical parameters (triglycerides [TG], low‐density lipoprotein [LDL]‐C, HLD‐C, cholesterol, alanine aminotransferase [ALT], aspartate aminotransferase [AST], and alkaline phosphatase [ALP]), and histopathology of liver tissue in a high‐fat diet (HFD)‐induced animal model of MASLD.

**Table 1:** Previously reported phytochemical characterization of the investigated medicinal plants and their relevance to MASLD (metabolic dysfunction‐associated steatotic liver disease)‐related mechanisms.

Intervention	Major phytochemical constituents reported in previous studies	Representative analytical methods reported in literature	Principal biological activities relevant to MASLD
Barberry (*Berberis vulgaris*/*Berberis* spp.) [19, 20]	Berberine, palmatine, berbamine, jatrorrhizine, oxyacanthine, catechin, gallic acid, caffeic acid, ferulic acid, luteolin, resveratrol, anthocyanins, organic acids (malic, citric, and tartaric acids)	HPLC‐UV, RP‐HPLC, HPLC‐DAD, LC‐MS phytochemical profiling, alkaloid and phenolic characterization studies	Activation of AMPK signaling, inhibition of hepatic lipogenesis (SREBP‐1c), improvement of lipid metabolism, lipid‐lowering, antioxidant (↓ROS, ↑SOD/GPx), suppression of NF‐κB and proinflammatory cytokines, hepatoprotection [13, 21, 22]
Jujube (*Ziziphus jujuba* Mill.) [23–26]	Polyphenols, flavonoids, catechin, rutin, quercetin derivatives, gallic acid, chlorogenic acid, betulinic acid, oleanolic acid, ursolic acid, polysaccharides, free amino acids, reducing sugars, vitamin C	HPLC, HPLC‐DAD, physicochemical and biochemical characterization, polyphenol profiling studies	Strong antioxidant and free radical scavenging activity, inhibition of lipid peroxidation (↓MDA), immunomodulation, attenuation of inflammatory cytokine production, improvement of glucose and lipid metabolism, hepatoprotection [27, 28]
Flixweed (*Descurainia sophia* L.) [29–32]	Quercetin, isorhamnetin, isoquercitrin, kaempferol derivatives, sinapic acid, glucosinolates (sinigrin, gluconapin), polysaccharides, mucilage fractions, daucosterol, helveticoside, and related cardiac glycosides	HPLC‐DAD, HPLC fingerprinting, chemometric characterization, metabolomics‐based analyses	Antioxidant activity, anti‐inflammatory effects, modulation of gut–liver axis function, regulation of intestinal barrier integrity, attenuation of oxidative stress and metabolic inflammation [9, 33]
Livergol (milk thistle; *Silybum marianum*) [34–36]	Silymarin complex (silybin A/B, isosilybin A/B, silychristin, isosilychristin, silydianin), taxifolin, flavonoids, phenolic acids, unsaturated fatty acids	HPLC‐UV, HPLC‐MS/MS, UHPLC‐MS/MS, flavonolignan standardization studies	Hepatoprotection, enhancement of antioxidant defense systems, activation of Nrf2 signaling, suppression of NF‐κB activation, stabilization of hepatocyte membranes, improvement of insulin sensitivity and lipid metabolism [16, 37, 38]

*Note*: Data compiled from peer‐reviewed studies using comparable hydroalcoholic (70% ethanol) extraction protocols [20, 23–26, 29–32, 35–37]. Direct reanalysis of the current batches was not performed, as the focus was on comparative in vivo efficacy. These established profiles provide strong mechanistic support for the observed hepatoprotective, antioxidant, and anti‐inflammatory effects.

## 2. Materials and Methods

### 2.1. Materials and Diet

Livergol was obtained from the Goldaru Company (Tehran, Iran). The fruits of jujube, barberry, and flixweed were collected from local farmers and authenticated by a botanist at Ferdowsi University of Mashhad (barberry: E‐1648 FUMA; jujube: E‐1649 FUMA; flixweed: E‐1650 FUMA). The HFD was formulated to contain 60% of energy from fat, 20% of energy from protein, and 20% of energy from carbohydrate, with a total energy density of 5.21 kcal/g and was purchased from New Jersey (Catalog Number D12492, New Brunswick, New Jersey, United States). The standard chow diet (SCD) contained 6.2% of energy from fat, 18% crude protein, 45% of energy from carbohydrate, and 4% crude fiber, with a total energy density of 3.1 kcal/g and was obtained from Madison (Catalog Number Teklad 7013, Madison, Wisconsin, United States). All macronutrient percentages reported refer to their contribution to the total energy intake rather than weight‐based composition.

### 2.2. Extraction

To prepare hydroalcoholic extracts, the fruits were first dried in the shade at room temperature until reaching a constant weight. They were then ground using an electric grinder and sieved through a suitable mesh. For extraction, 70% ethanol (ethanol:water, 70:30 v/v) was used as the solvent at a 1:10 (w/v) ratio. A specified amount of the dried plant powder was macerated in the specified solvent for 72 h at room temperature in the dark, with the mixture being stirred every 8–12 h to ensure complete contact between the solid and liquid phases. After the extraction, the solutions were filtered through a Whatman No. 1 filter paper, and the plant residue was extracted again with a fresh solvent to increase the final yield. The extracts from each plant were concentrated under reduced pressure at temperatures below 45°C using a rotary evaporator to remove the solvent.

Subsequently, the concentrated extracts were freeze‐dried to remove any remaining moisture, resulting in the final powdered extracts. The dried extracts were stored in dark containers at 4°C until use. The extraction yield was calculated as the percentage of the dry extract weight relative to the initial powder weight [39–41]. To prepare the animal dosing solution, the extracts were dissolved in distilled water according to the predetermined doses (jujube 400 mg/kg, barberry 100 mg/kg [42], and flixweed 100 mg/kg [9]) and mixed thoroughly before administration to ensure uniformity.

### 2.3. Experimental Design

Forty‐eight male Wistar rats (6–8 weeks old; weighing 250 ± 10 g) were obtained from the Experimental Studies and Laboratory Animals Center of Birjand University of Medical Sciences. The animals were housed under standard laboratory conditions, including a controlled temperature (25 ± 2°C), a 12 h light/12 h dark cycle (lights on at 07:00 a.m. and off at 07:00 p.m.), humidity (25%–35%), and free access to food and water, in accordance with the ethical principles for the care and use of laboratory animals.

In this preclinical study, after a 1‐week adaptation period, the animals were randomly divided into six groups (*n* = 8) using a block randomization method: Group 1: normal rats as healthy controls; Group 2: MASLD rats induced by a HFD as the disease control; Group 3: MASLD rats treated with barberry extract (100 mg/kg body weight); Group 4: MASLD rats treated with jujube extract (400 mg/kg body weight); Group 5: MASLD rats treated with flixweed extract (100 mg/kg body weight); Group 6: MASLD rats treated with Livergol (200 mg/kg body weight). The selected doses of barberry, jujube, flixweed, and Livergol were determined based on previously published studies [8, 43–46].

All doses were calculated based on the body weight of each animal (mg/kg) and were adjusted weekly according to the most recent body weight measures to ensure accurate and consistent dosing throughout the intervention period. The treated groups received their respective interventions orally by gavage once daily between 09:00 and 10:00 a.m., corresponding to the early light phase of the circadian cycle and prior to the peak feeding period, for eight consecutive weeks. This standardized timing was selected to minimize potential confounding effects related to the circadian rhythm and food intake on metabolic outcomes. The control groups (Groups 1 and 2) received an equivalent volume of distilled water by gavage during the same period.

### 2.4. Body Weight Measurement

The body weight of all animals was recorded throughout the experimental period to monitor physiological changes associated with MASLD induction and treatment. Rats were weighed at baseline (prior to dietary intervention) and then once weekly until the end of the intervention (8 weeks) using a calibrated digital balance with a precision of 0.01 g. All measurements were performed in the morning (08:00–10:00) to minimize circadian variations.

### 2.5. Relative Food Intake Assessment

Food intake was assessed to evaluate dietary consumption and its potential influence on metabolic outcomes. The amount of food provided to each cage was weighed daily, and the remaining food was measured 24 h later. The daily food intake for each rat was calculated by subtracting the leftover food from the initial amount provided.

Daily food intake g/day= initial food provided g−remaining food after 24 h g.

To account for differences in body size, food intake was normalized and expressed as follows:

Relative food intake food [g]/100 g body weight/day=food intake g/day÷body weight g×100.

For normalization, the mean body weight of rats within each cage was used to calculate the relative food intake values. All measurements were conducted under controlled environmental conditions.

### 2.6. Sample Collection

At the end of the 8 week intervention, the animals were anesthetized by an intraperitoneal injection of ketamine (50 mg/kg) and xylazine (10 mg/kg). An adequate depth of anesthesia was confirmed by the absence of pedal withdrawal and corneal reflexes. Animals were subsequently euthanized by exsanguination via cardiac puncture under deep anesthesia, followed by cervical dislocation to ensure complete and irreversible cessation of vital functions in accordance with institutional ethical guidelines for animal care and use. Following laparotomy, blood samples were collected directly from the heart, and the obtained serum was separated and stored for biochemical and oxidative stress analyses. The liver was then excised and rinsed with normal saline. A portion of the liver tissue was fixed in 10% formalin for histopathological examinations, while another portion was immediately transferred to liquid nitrogen for subsequent molecular analyses.

### 2.7. Biochemical Parameters

Serum levels of liver function enzymes (ALT, AST, and ALP) and lipid profile (LDL, high‐density lipoprotein [HDL], TG, total cholesterol [Chol]) components were measured using commercial diagnostic kits (Pars Azmoon Co., Tehran, Iran) and analyzed with an automated biochemical analyzer (Prestige, Japan).

### 2.8. Oxidative Stress Parameter Assays

Serum samples were thawed at 4°C and gently mixed before the analysis.

MDA, as a marker of lipid peroxidation, was quantified using the thiobarbituric acid (TBA) reactive substances (TBARS) assay. Briefly, serum aliquots were incubated with TBA reagent under acidic conditions and heated at 90–100°C for 60 min. After cooling, the absorbance of the MDA–TBA adduct was measured spectrophotometrically at 532–535 nm using a microplate reader [47].

TAC was evaluated by the automated ABTS assay, in which the ABTS^+^ radical cation was reduced by antioxidants present in the sample. The decrease in absorbance at 660 nm was proportional to the antioxidant capacity, and results were expressed as mmol Trolox equivalent per liter [48].

Total thiol (–SH) group content, as an indicator of nonenzymatic antioxidant capacity, was determined using the Ellman’s reagent (5,5′‐dithiobis(2‐nitrobenzoic acid), DTNB) assay. In brief, serum samples were incubated with DTNB in 0.1 M phosphate buffer (pH 8.0) at room temperature for 15 min, allowing the formation of the yellow‐colored 5‐thio‐2‐nitrobenzoic acid (TNB) complex. The absorbance of the resulting TNB complex was measured at 412 nm using a spectrophotometer, and the total thiol content was calculated based on a standard curve [47, 48].

### 2.9. Gene Expression Assay

To evaluate the expression levels of target genes (IL‐1β, IL‐6, IL‐10, and TNF‐α) in the liver tissue, samples were ground in a mortar under liquid nitrogen. Total RNA was extracted from ~50–100 mg of liver tissue using an RNA extraction kit (Anacell, Iran) according to the manufacturer’s instructions. The purity and concentration of the extracted RNA were determined using a NanoDrop spectrophotometer at wavelengths of 260 and 280 nm, and samples with an A260/A280 ratio between 1.8 and 2.0 were selected for further analysis.

For cDNA synthesis, 1 µg of total RNA was used as a template with a cDNA synthesis kit (Anacell, Iran) following the manufacturer’s protocol. Quantitative real‐time PCR (qRT‐PCR) was performed to determine the expression levels of the target genes using a real‐time PCR system (StepOne, Applied Biosystems) and a SYBR Green master mix. Each reaction was carried out in a total volume of 20 µL containing 10 µL SYBR Green master mix, 1 µL of each primer (forward and reverse, 10 pmol), 2 µL cDNA, and 6 µL nuclease‐free water. The thermal cycling conditions consisted of an initial denaturation at 95°C for 10 min, followed by 40 cycles of denaturation at 95°C for 15 s and annealing/extension at 60°C for 60 s. A melting curve analysis was performed at the end of the amplification to verify the specificity of the PCR products.

The GAPDH gene was used as the internal housekeeping control for normalization, and relative gene expression levels were calculated using the 2^–ΔΔCt^ method. All reactions were performed in duplicate. Table 2 shows the primer sequences of IL‐1β, IL‐6, IL‐10, TNF‐α, and GAPDH genes.

**Table 2:** Primer sequences for real‐time PCR.

Genes	Primer sequence
GAPDH	F: 5′‐TCATCAACGGCACAGTCAAGG‐3′
	R: 5′‐TTCTGCATGGTGGTGAAGACG‐3′
IL‐1β	F: 5′‐TGAGCTTTCGACAGTGAGGAG‐3′
	R: 5′‐TAGTCGAGATGCTGCTGTGAG‐3′
IL‐6	F: 5′‐TCATACCACCCACAACAGACC‐3′
	R: 5′‐TCTCTGACAGTGCATCATCGC‐3′
IL‐10	F: 5′‐TCCACTTCCCAGTCAGCCAG‐3′
	R: 5′‐TCACCCAAGTAACCCTTAAAGTCC‐3′
TNF‐α	F: 5′‐TGAACTTCGGGGTGATCGGTC‐3′
	R: 5′‐TTGGTGGTTTGCTACGACGTG‐3′

Abbreviations: GAPDH, glyceraldehyde‐3‐phosphate dehydrogenase; IL‐1β, interleukin‐1 beta; IL‐6, interleukin‐6; IL‐10, interleukin‐10; TNF‐α, tumor necrosis factor‐alpha.

### 2.10. Histopathological Examination of Liver Tissue

To assess histological changes in the liver, samples collected from corresponding liver lobes were immediately fixed in 10% buffered formalin for at least 48 h following euthanasia. After fixation, the samples underwent routine processing, including dehydration through a graded ethanol series (70%, 80%, 90%, 96%, and 100%), clearing with xylene, and paraffin infiltration. The resulting paraffin blocks were sectioned at a thickness of 5 µm using a microtome, and the sections were mounted onto glass slides. The sections were then stained with hematoxylin and eosin (H&E) and examined under a light microscope (Olympus, Japan). Stained slides were examined under a light microscope at various magnifications to assess the hepatic architecture, hepatocyte morphology, inflammatory cell infiltration, and other pathological changes. The steatosis score (ballooning) assessed the quantities of large or medium‐sized lipid droplets, from grade 0–3 (0: <5%; 1: 5%–33%, 2: 34%–66%, and 3: >67%, marked). Other hepatological injuries were assessed according to the studies by Bedossa et al. [49] and Brunt et al. [50] studies. Histological changes were evaluated by a pathologist who was blinded to the experimental groups.

### 2.11. Statistical Analysis

All data were first assessed for normality using the Shapiro–Wilk test. Quantitative values are presented as the mean ± standard deviation (mean ± SD). Comparisons among multiple groups were performed using one‐way analysis of variance (one‐way ANOVA), and when a significant difference was detected, Tukey’s post hoc test was applied for pairwise comparisons between groups. Differences with a *p*‐value < 0.05 were considered statistically significant. All statistical analyses and graphing were performed using GraphPad Prism software, version 10.

## 3. Results

### 3.1. Body Weight Changes and Relative Food Intake

An increasing trend in body weight was observed in all groups; however, the magnitude of these changes differed significantly between the treated and control groups. The MASLD group experienced the highest weight gain and showed a statistically significant difference compared to the Normal group from the first week to the end of the study (*p* < 0.05), reaching 422.9 g by week 8. In contrast, treatment with plant extracts and Livergol effectively controlled body weight in MASLD rats. The Barberry, Jujube, and Livergol groups showed significant reductions in body weight relative to the MASLD group starting from weeks 2, 4, and 3, respectively. Notably, in the final weeks, barberry exhibited a significantly superior effect on weight control compared to the other treatments (*p* < 0.05). Although the flixweed group also had lower body weight than the disease control, its effect was weaker than that of barberry and jujube.

Analysis of relative food intake (grams of food per 100 g of body weight) revealed an inverse relationship between body weight and appetite. Despite the greatest weight gain, the MASLD group consumed the least food throughout the study, particularly from week 2 onward, compared to the Normal group (*p* < 0.05). Treatment with barberry and jujube significantly (*p* < 0.05) modulated food intake relative to the MASLD group, bringing it closer to the Normal group level. Livergol also showed a significant difference from the MASLD group from week 3 onward (*p* < 0.05). In this measure, the Barberry and Jujube groups maintained a more stable and healthy feeding pattern compared to Livergol and Flixweed. Table 3 shows the changes in body weight and relative food intake in the study groups.

**Table 3:** Weight and relative food intake of the study groups during the intervention.

Variables		Normal	MASLD	Barberry	Jujube	Flixweed	Livergol
Body weight (g)	W0	245.57 ± 8.3	250.1 ± 18.3	249 ± 7.5	255.6 ± 12.1^&^	247.8 ± 8.8	253.9 ± 7.9
	W1	264.1 ± 6.8	278.1 ± 9.28^∗^	266.5 ± 10.37	259.6 ± 7.11^#&^	273.5 ± 9.6	268.2 ± 9.3
	W2	280 ± 5.9	302.3 ± 5.82^∗^	278.7 ± 6.25^#&^	287.6 ± 13.3^#&^	298.5 ± 11.56^∗^	289.5 ± 6.29
	W3	284.2 ± 10.4	314.5 ± 6.8^∗^	298.6 ± 12.3^∗^^#^	302.6 ± 8.03^∗^	311.9 ± 3.4^∗^	296.2 ± 11.5^#&^
	W4	298 ± 6.8	342.9 ± 11.5^∗^	311.4 ± 10.9^∗^^#&$^	313.1 ± 7.1^∗^^#&$^	340 ± 5.52^∗^	328 ± 9.2^∗^^#^
	W5	310.4 ± 8.17	368 ± 6.5^∗^	320 ± 9.8^#&$^	328.6 ± 10.2^∗^^#&^	362.4 ± 9.7^∗^	337.5 ± 11.23^∗^^#&^
	W6	319.5 ± 12.8	375.5 ± 10.35^∗^	340.4 ± 7.8^∗^^#&^	349 ± 4.94^∗^^#&^	370 ± 4.6^∗^	354.8 ± 14.35^∗^^#&^
	W7	335.2 ± 7.5	405 ± 12^∗^	353.9 ± 11.9^∗^^#&^	366 ± 11.37^∗^^#^	379.2 ± 11.85^∗^^#^	369.6 ± 7.6^∗^^#^
	W8	347.4 ± 9.12	422.9 ± 7.38^∗^	366.5 ± 8.7^∗^^#&$^	380 ± 8^∗^^#&^	403.3 ± 8.82^∗^^#^	387.8 ± 13.26^∗^^#&^
Food intake (food (g)/100 g body weight/day)	W1	7.7 ± 0.3	7.1 ± 1	7.2 ± 0.47	7.4 ± 0.27	7.2 ± 0.82	7 ± 0.28
	W2	7.24 ± 0.52	6 ± 0.34^∗^	6.89 ± 0.39^#^	6.82 ± 0.59^#^	6.35 ± 0.53^∗^	6.34 ± 0.31^∗^
	W3	7 ± 0.31	5.61 ± 0.43^∗^	6.07 ± 0.44^∗^	6.15 ± 0.35^∗^	5.95 ± 0.12^∗^	6.56 ± 0.48^#&^
	W4	6.59 ± 0.34	5.1 ± 0.26^∗^	6.19 ± 0.26^#&^	6 ± 0.52^∗^^#&^	5.36 ± 0.24^∗^	5.77 ± 0.25^∗^^#^
	W5	6.32 ± 0.41	4.78 ± 0.29	6.02 ± 0.2^∗^^#&^	5.88 ± 0.36^#&^	5.1 ± 0.4^∗^	5.94 ± 0.38^#&^
	W6	6.16 ± 0.39	4.4 ± 0.41^∗^	5.42 ± 0.3^∗^^#&^	5.45 ± 0.16^∗^^#&^	4.83 ± 0.26^∗^	5.43 ± 0.28^∗^^#&^
	W7	5.9 ± 0.29	4.26 ± 0.24^∗^	5.38 ± 0.42^∗^^#&^	5.18 ± 0.31^∗^^#&^	4.65 ± 0.33^∗^	5.15 ± 0.32^∗^^#&^
	W8	5.64 ± 0.38	3.95 ± 0.17^∗^	5.11 ± 0.26^∗^^#&^	5.12 ± 0.32^∗^^#&^	4.32 ± 0.25^∗^	4.89 ± 0.25^∗^^#&^

*Note*: Data are presented as mean ± SD.

Abbreviation: W, week.

^∗^ = Significant difference with the Normal group (*p* < 0.05).

^#^ = Significant difference with the MASLD (metabolic dysfunction‐associated steatotic liver disease) group (*p* < 0.05).

^&^ = Significant difference with the Flixweed group (*p* < 0.05).

^$^ = Significant difference with the Livergol group (*p* < 0.05); *n* = 8.

### 3.2. Serum Lipid Profile

The serum TG level in the MASLD group showed a significant increase of ~89.4% compared to the normal group (*p* < 0.0001). However, in comparison with the MASLD group, the treatment groups receiving barberry, jujube, and livergol showed significant decreases in TG levels by ~32.0%, ~26.9%, and ~31.0%, respectively (*p* < 0.0001).

The serum Chol level in the MASLD group increased significantly by ~51.2% compared to the normal group (*p* < 0.0001). Relative to the MASLD group, the treatment groups barberry, jujube, and livergol showed significant reductions of ~17.6%, ~12.9%, and ~11.3%, respectively (*p* < 0.001).

Compared to the normal group, the serum LDL level in the MASLD group significantly increased by ~83.3% (*p* < 0.0001). In contrast, the Jujube‐treated group showed the greatest significant reduction in LDL level (~34.1%) compared to the MASLD group (*p* < 0.0001). The Barberry and Livergol groups also showed significant decreases of ~27.3% and ~22.7%, respectively, compared with the MASLD group (*p* < 0.0001).

The serum HDL level in the MASLD group (19.5 mg/dL) was significantly lower than that of the normal group (32.75 mg/dL), representing a decrease of ~40.9% (*p* < 0.0001). Compared to the MASLD group, the Barberry (22 mg/dL) and Livergol (23.75 mg/dL) groups showed significant increases in HDL levels, corresponding to ~12.8% and 23.38% increases, respectively (*p* < 0.005).

In the Flixweed‐treated group, compared to the MASLD group, serum TG, LDL, and Chol levels decreased by ~0.89%, 3.69%, and 6.8%, respectively, while serum HDL increased by about 9.61%; however, these changes were not statistically significant. Table 4 shows the serum TG, LDL, HDL, and cholesterol levels in the study groups.

**Table 4:** The effect of barberry, jujube, flixweed, and livergol on serum lipid profile levels (TG, LDL, HDL, and cholesterol).

Parameters	Normal	MASLD	Barberry	Jujube	Flixweed	Livergol
TG (mg/dL)	51.7 ± 2.5^∗^	96.1 ± 6.6	67.7 ± 3.8^∗^	72.5 ± 2.2^∗^	95.2 ± 5.6	67.5 ± 3.1^∗^
LDL (mg/dL)	11.8 ± 1.8^∗^	22 ± 2.8	16.2 ± 2.1^∗^	14.5 ± 1.3^∗^	20.5 ± 1.7	17.1 ± 1.4^∗^
HDL (mg/dL)	32.7 ± 1.8^∗^	19.2 ± 1.6	22 ± 2.5^∗^	20.6 ± 1.5	21.1 ± 2.2	23.7 ± 1.6^∗^
Cholesterol (mg/dL)	40.7 ± 2.7^∗^	61.8 ± 4.5	51.25 ± 2.7^∗^	54.5 ± 3.3^∗^	59.6 ± 4.9	54.5 ± 2.8^∗^

*Note*: Data are presented as mean ± SD.

Abbreviations: HDL, high‐density lipoprotein; LDL, low‐density lipoprotein; MASLD, metabolic dysfunction‐associated steatotic liver disease; TG, triglycerides.

^∗^ = Significant difference with the MASLD group (*p* < 0.05); *n* = 8.

### 3.3. Liver Function Enzymes

In the MASLD group, serum levels of AST, ALT, and ALP were significantly elevated compared to those of the normal group, with increases of ~142.9%, ~91.4%, and ~66.7%, respectively (*p* < 0.0001).

Following the therapeutic interventions, the Barberry group showed the best performance in reducing the levels of both AST and ALT compared to those of the other treatments. In the Barberry group, AST levels were significantly reduced to 141.2 U/L and ALT levels to 138.9 U/L, representing reductions of ~16.5% and 21.9%, respectively, compared to those of the MASLD group (*p* < 0.0001).

The Jujube group also resulted in significant reductions in the serum levels of both transaminases, with AST reduced by ~12.9% and ALT reduced by ~17.4% compared to the MASLD group (*p* < 0.0001). The Livergol group reduced AST to 133.7 U/L (a reduction of about 19.5%) and ALT to 165.4 U/L (a reduction of about 7.2%); however, only the decrease in AST was statistically significant compared to the MASLD group (*p* < 0.0001). In contrast, the Flixweed group showed no significant reduction, and the enzyme levels did not differ significantly from those in the MASLD group.

When evaluating the effects of treatments on ALP, the reductions were relatively smaller compared to those of transaminases. The Barberry and Jujube groups reduced ALP to 116.8 U/L and 116.5 U/L, respectively, with significant reductions of about 8.0% and 8.8% compared to the MASLD group (*p* = 0.0282). The Livergol group also showed a significant decrease of about 8.0% compared to the MASLD group (*p* <0.0111), while the Flixweed group showed only a slight difference compared to the MASLD group. Table 5 shows the serum levels of liver function enzymes (AST, ALT, and ALP) in the study groups.

**Table 5:** The effect of barberry, jujube, flixweed, and livergol on serum levels of liver function enzymes (AST, ALT, and ALP).

Parameters	Normal	MASLD	Barberry	Jujube	Flixweed	Livergol
AST (U/L)	71.5 ± 4.3^∗^	170.8 ± 11.6	141.2 ± 6.7^∗^	148.6 ± 9.8^∗^	161 ± 8.8	133.7 ± 9.4^∗^
ALT (U/L)	92.4 ± 2.9^∗^	178.3 ± 5.2	138.9 ± 1.1^∗^	146.6 ± 3.6^∗^	174 ± 10.23	165.4 ± 3^∗^
ALP (U/L)	79 ± 6.5^∗^	124.8 ± 8	116.8 ± 4.3^∗^	116.5 ± 3.2^∗^	123.7 ± 5.2	115.8 ± 4.7^∗^

*Note*: Data are presented as mean ± SD.

Abbreviations: ALP, alkaline phosphatase; ALT, alanine aminotransferase; AST, aspartate aminotransferase; MASLD, metabolic dysfunction‐associated steatotic liver disease.

^∗^ = Significant difference with the MASLD group (*p* < 0.05); *n* = 8.

### 3.4. Oxidative Stress Markers

**Figure 1:**
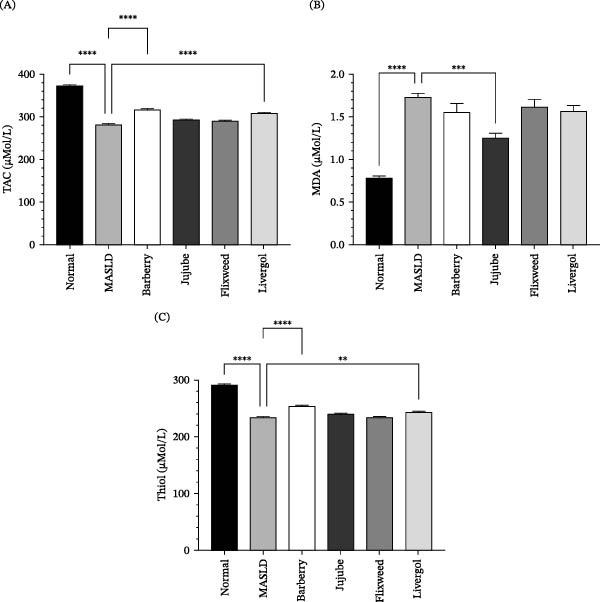
Effect of barberry, jujube, flixweed, and livergol on serum levels of (A) total antioxidant capacity (TAC); (B) malondialdehyde (MDA); (C) thiol; *n* = 8;  ^∗∗^ = *p* < 0.01;  ^∗∗∗^ = *p* < 0.001;  ^∗∗∗∗^ = *p* < 0.000, MASLD: metabolic dysfunction‐associated steatotic liver disease.

The serum level of TAC (Figure 1A) was 281.5 μMol/L in the MASLD group, showing a ~25.3% significant decrease compared to the normal group (371.4 μMol/L) (*p* < 0.0001). The treatments exhibited different effects on the TAC levels. The Barberry and Livergol groups showed significant increases in serum TAC compared to the MASLD group (*p* < 0.0001). Barberry demonstrated the highest increase, ~12.5%, while Livergol showed an increase of ~10.0%. The Jujube and Flixweed groups exhibited a slight, nonsignificant increase compared to the MASLD group.

Serum MDA levels (Figure 1B) in the MASLD group were significantly elevated by ~118.8% compared to those in the normal group (*p* < 0.0001). Treatment with the extracts led to a reduction in serum MDA levels relative to the MASLD group, but this reduction was statistically significant only in the Jujube group, which showed a ~25.8% decrease (*p* < 0.0001).

The serum thiol level (Figure 1C) in the MASLD group (233.1 μMol/L) showed a significant ~22.0% reduction compared to the Normal group (290.7 μMol/L) (*p* < 0.0001). In the treatment groups, serum thiol levels significantly increased by ~8.71% in the Barberry group and ~4.76% in the Livergol group compared to the MASLD group (*p* < 0.0001, and*p* = 0.0038, respectively).

### 3.5. Expression of Pro‐ and Anti‐Inflammatory Cytokine Genes

**Figure 2:**
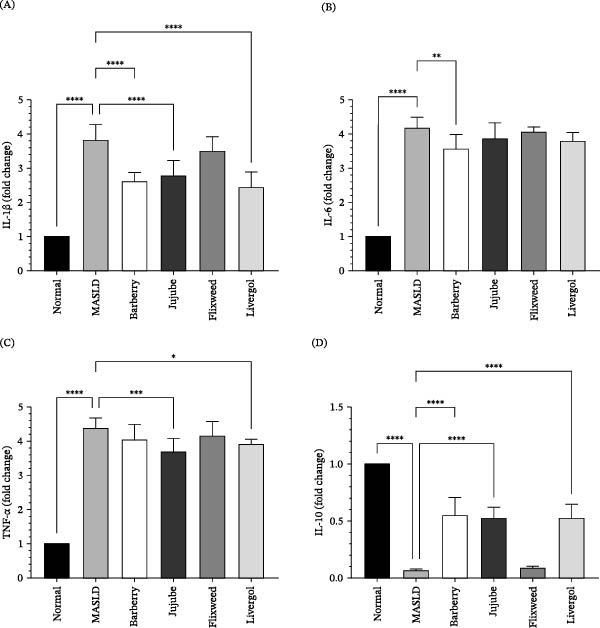
Effect of barberry, jujube, flixweed, and livergol on (A) interleukin‐1 beta (IL‐1β); (B) interleukin‐6 (IL‐6), (C) tumor necrosis factor‐alpha (TNF‐α); (D) interleukin‐10 (IL‐10). *n* = 8.  ^∗^ = *p* < 0.05,  ^∗∗^ = *p* < 0.01,  ^∗∗∗^ = *p* < 0.001,  ^∗∗∗∗^ = *p* < 0.0001, MASLD: metabolic dysfunction‐associated steatotic liver disease.

In the MASLD group, the expression levels of proinflammatory cytokine genes IL‐1β, IL‐6, and TNF‐α were significantly upregulated compared to the Normal group (*p* < 0.0001), with increases of ~283.3%, ~311.1%, and ~325%, respectively (Figure 2A–C).

In response to therapeutic interventions, the Barberry and Jujube groups exhibited the most pronounced reductions in the expression of these cytokine genes. Specifically, IL‐1β expression levels were significantly decreased by ~40.2%, ~38.1%, and ~45.6% in the Barberry, Jujube, and Livergol groups, respectively, compared to the MASLD group (*p* < 0.0001) (Figure 2A).

The expression of IL‐6 was reduced in all treatment groups compared to the MASLD group; however, this decrease was statistically significant only in the Barberry group, showing a reduction of ~22.2% (*p* < 0.001) (Figure 2B).

Similarly, TNF‐α expression was significantly decreased in the Jujube and Livergol groups compared to the MASLD group (*p* < 0.05), with reductions of ~12.9% and ~5.9%, respectively (Figure 2C).

Conversely, the expression of the anti‐inflammatory cytokine IL‐10 was significantly downregulated (by about 95%) in the MASLD group compared to that in the normal group (*p* < 0.0001). Treatment with Barberry, Jujube, and Livergol significantly increased IL‐10 gene expression compared to the MASLD group (*p* < 0.0001). Among the treatments, barberry showed the most pronounced effect in enhancing IL‐10 expression, while Jujube and livergol demonstrated a similar effect (Figure 2D).

Treatment with Flixweed did not produce any statistically significant changes in the expression of either pro‐ or anti‐inflammatory cytokine genes. Overall, Flixweed treatment slightly reduced the expression of IL‐1β, IL‐6, and TNF‐α, and modestly increased IL‐10 expression compared to the MASLD group (nonsignificant). Figure 2 illustrates the relative expression levels of IL‐1β, IL‐6, IL‐10, and TNF‐α genes.

### 3.6. Histopathological Evaluation of Liver Tissue

**Figure 3:**
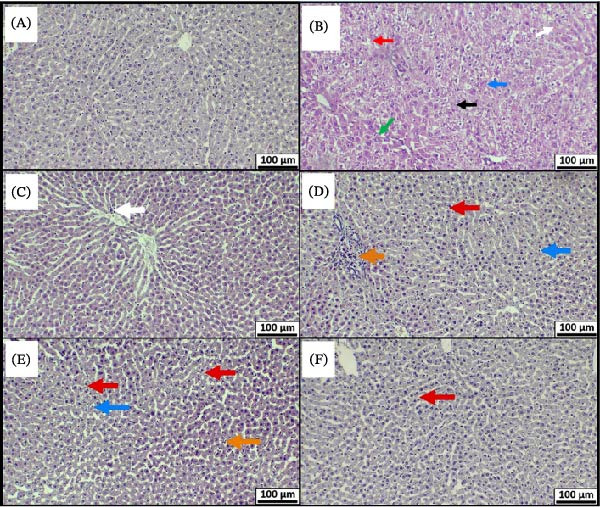
Representative histopathological images of liver tissue in (A) normal; (B) metabolic dysfunction‐associated steatotic liver disease (MASLD); (C) barberry, (D) jujube, (E) flixweed, and (F) livergol‐treated groups. Hematoxylin and eosin staining. 400x. Ballooning: red arrow, necrosis: blue arrow, bile duct hyperplasia: green arrow, grade 3 inflammation: black arrow, grade 2 inflammation: orange arrow, grade 1 inflammation: white arrow.

The histopathological examination of liver tissues revealed marked differences among the experimental groups. In the normal control group, the hepatic architecture appeared completely intact with no observable pathological alterations (Figure 3A). In contrast, the MASLD group exhibited severe hepatic injuries, including grade 3 inflammation, grade 2 necrosis, grade 2 hepatocellular ballooning, and bile duct hyperplasia. A pronounced accumulation of inflammatory cells was also observed, indicating the substantial progression of hepatic damage (Figure 3B).

In the groups treated with herbal extracts, remarkable improvements were observed in histopathological indices. In the Barberry group, the severity of inflammation was reduced to grade 1, with only a mild increase in inflammatory cell infiltration, reflecting a notable anti‐inflammatory effect of the extract (Figure 3C). In the Jujube group, the severity of inflammation and necrosis decreased to grades 2 and 1, respectively, and hepatocellular ballooning was reduced to grade 1, although inflammatory cell accumulation was still noticeable (Figure 3D). The Flixweed group showed similar tissue alterations with necrosis and ballooning (both grade 1) and grade 2 inflammation, but the overall improvement was less pronounced compared with that of other treatments (Figure 3E). In the Livergol group, hepatocellular ballooning was reduced to grade 1, accompanied by only a slight increase in the inflammatory cell count (Figure 3F).

Overall, histopathological findings demonstrated that all therapeutic interventions partially improved hepatic structure and alleviated MASLD‐induced damage. However, the most significant protective and restorative effects were observed in the groups treated with hydroalcoholic extracts of barberry and livergol, both of which markedly reduced hepatic inflammation and pathological cellular alterations. Figure 3 shows the histopathological features of liver tissue in the studied groups. Table 6 reports the semiquantitative histopathological evaluation of liver tissue.

**Table 6:** Semiquantitative histopathological evaluation of liver tissue.

Parameters	Normal	MASLD	Barberry	Jujube	Flixweed	Livergol
Inflammation	0	3	1	2	2	1
Hepatocyte ballooning	0	2	0	1	1	1
Necrosis	0	2	0	1	1	0

## 4. Discussion

### 4.1. Main Findings

The results of the present study demonstrated that induction of MASLD in the animal model led to significant disturbances in the lipid profile, relative food intake, body weight gain, elevated liver enzymes, exacerbated oxidative stress, upregulation of inflammatory gene expression, and severe histopathological damage in liver tissue. In this context, intervention with hydroalcoholic extracts of barberry, jujube, and livergol significantly ameliorated many of these abnormalities, whereas flixweed extract exhibited only limited therapeutic effects.

The findings of the present study showed an inverse relationship between body weight and relative food intake, with the MASLD group having the greatest weight gain but the lowest food intake relative to body weight. This seemingly paradoxical phenomenon has been well documented in HFD‐induced obesity models and may be attributed to the high energy density of HFDs, whereby animals consume fewer grams of food while ingesting a greater caloric load [51]. In addition, leptin resistance and impaired satiety signaling in obese rodents may contribute to an apparent reduction in food intake per unit body weight [52]. Berberine, the major bioactive constituent of barberry, exerts potent antiobesity effects. Previous experimental studies have suggested that these effects may be associated with activation of AMP‐activated protein kinase (AMPK), enhancement of fatty acid oxidation, suppression of hepatic lipogenesis, and improvement of insulin sensitivity; however, these pathways were not directly evaluated in the present study [8, 53]. Jujube mediates its metabolic effects via bioactive compounds such as lupeol and betulinic acid. Evidence from previous studies indicates that these compounds may modulate PI3K/Akt and AMPK signaling pathways, improve insulin sensitivity, inhibit lipogenesis, and attenuate HFD‐induced weight gain, although these mechanisms were not investigated in the current experiment [11, 54]. Silymarin, the principal active component of livergol, also exhibits antiobesity properties. Earlier studies have proposed that these effects may involve stimulation of adipose tissue thermogenesis, enhancement of mitochondrial biogenesis, modulation of FXR signaling, and suppression of NF‐κB activity; however, direct assessment of these pathways is beyond the scope of the present study [55, 56]. Clinical evidence has confirmed that silymarin supplementation leads to significant reductions in body mass index, epididymal adipose tissue weight, and improvements in lipid profiles in obese individuals [55–57]. The improvement in relative food intake observed in the treated groups—particularly those receiving barberry and jujube extracts—suggests a regulatory effect of these interventions on appetite control mechanisms and energy metabolism. Previous studies have reported that these effects are likely mediated through enhanced leptin signaling, modulation of appetite‐related neurotransmitters such as serotonin, and regulation of genes involved in energy homeostasis [58, 59].

In the present study, induction of MASLD resulted in a significant increase in serum TG (~89.4%) and LDL‐C (~83.3%) and a marked decrease in HDL‐C (~40.9%), consistent with findings from previous studies in similar animal models [60]. Barberry extract demonstrated the strongest lipid‐lowering effect, reducing TG by ~32% and LDL‐C by ~27.3%, which can be attributed to the berberine alkaloid present in this plant. Multiple studies have shown that berberine reduces lipid synthesis and enhances fatty acid oxidation through activation of AMPK and inhibition of sterol regulatory element‐binding protein‐1c (SREBP‐1c) [53]. Furthermore, the significant increase in HDL‐C observed in the Barberry (~12.8%) and Livergol (~23.3%) groups indicates a positive effect of these extracts on reverse cholesterol transport and improvement of the lipoprotein profile, in line with Zhao et al. [8], who reported berberine‐induced upregulation of HDL receptors. Jujube extract also exhibited notable hypolipidemic effects, with reductions of ~26.9% in TG and ~34.1% in LDL‐C, likely due to its high content of flavonoids and phenolic compounds, as reported in previous studies [45, 61].

In this study, induction of MASLD led to marked elevations in AST (~142.9%), ALT (~91.4%), and ALP (~66.7%), indicating severe hepatic damage and tissue inflammation. Barberry extract exhibited the strongest hepatoprotective effect, reducing AST by ~16.5% and ALT by ~21.9%, consistent with the findings of Zhu et al. [13], who reported that berberine protects hepatocytes by inhibiting apoptosis and decreasing ROS production. Jujube extract also significantly decreased both transaminases, which can be attributed to the potent antioxidant properties of its phenolic and flavonoid compounds. Studies have shown that these compounds can prevent hepatocyte membrane damage by inhibiting lipid peroxidation and enhancing endogenous antioxidant defense systems [62]. The relatively milder reduction in ALP compared to transaminases in the treatment groups likely indicates that canalicular injury and cholestasis were less pronounced than hepatocellular necrosis in this experimental model.

In the present study, a ~25.3% decrease in TAC and a ~118.8% increase in MDA levels in the MASLD group indicated exacerbated oxidative stress and lipid peroxidation in liver tissue. Barberry extract exhibited the strongest antioxidant effect, increasing TAC by ~12.5% and serum thiol levels by ~8.7%. Based on previous mechanistic studies, these effects may be related to activation of antioxidant defense pathways, including Nrf2/ARE signaling and increased expression of endogenous antioxidant enzymes [63]. Jujube extract showed the greatest inhibitory effect on lipid peroxidation, reducing MDA levels by ~25.8%, consistent with the findings of Liu et al. [54] regarding the potent antioxidant properties of jujube polysaccharides. The increase in serum thiol levels in the Barberry and Livergol groups indicates improved glutathione status and enhancement of the thiol‐dependent antioxidant defense system, which plays a vital role in neutralizing ROS and protecting cellular integrity [13, 16].

In our study, the expression levels of IL‐1β, IL‐6, and TNF‐α genes in the MASLD group increased by ~283.3%, ~311.1%, and ~325%, respectively, indicating a pronounced inflammatory response in liver tissue. Barberry and jujube extracts exhibited the strongest inhibitory effects on these genes, with barberry significantly reducing IL‐1β and IL‐6 expression. One possible explanation, supported by previous experimental evidence, is the modulation of NF‐κB‐dependent inflammatory signaling. Studies have shown that berberine can suppress proinflammatory cytokine expression by inhibiting IκB phosphorylation and preventing NF‐κB nuclear translocation [64]. Additionally, the reduction of TNF‐α expression in the Jujube and Livergol groups highlights the anti‐inflammatory properties of these extracts, consistent with Wei et al. [10], who reported inhibitory effects of Jujube polyphenols on inflammatory pathways. Conversely, a 95% decrease in IL‐10, a key anti‐inflammatory cytokine, in the MASLD group indicated impaired regulatory responses. The significant increase in IL‐10 expression in the Barberry, Jujube, and Livergol groups reflects the modulatory effects of these extracts in restoring the balance between pro‐ and anti‐inflammatory cytokines. Previous studies have suggested the potential involvement of pathways such as STAT3 signaling and regulatory T‐cell responses [11, 56, 65].

The histopathological results provided confirmatory evidence for the biochemical and molecular data, showing that MASLD induction led to severe hepatic tissue damage, including grade 3 inflammation, grade 2 necrosis, hepatocellular ballooning, and bile duct hyperplasia. These alterations resemble the histopathological pattern of NASH in humans, reflecting the progression from simple steatosis to the inflammatory stage of the disease [66]. Intervention with herbal extracts markedly improved liver tissue architecture, with barberry extract exhibiting the strongest protective effect by reducing inflammation to grade 1 and limiting inflammatory cell infiltration. These findings are consistent with Guo et al. [67], who reported berberine’s protective effects on liver histology in a NAFLD model. Jujube extract also significantly reduced necrosis and hepatocellular ballooning, likely due to the antiapoptotic and anti‐inflammatory properties of its bioactive compounds. Livergol demonstrated notable hepatoprotective effects by reducing hepatocellular ballooning and mild inflammation, which can be attributed to its phenolic components, including silymarin [57, 68].

Collectively, the beneficial effects observed in the present study may be explained by the phytochemical composition of the investigated interventions. Major marker compounds include berberine in barberry, polyphenols and triterpenic acids in jujube, quercetin/isorhamnetin derivatives and polysaccharides in flixweed, and silybin‐rich silymarin in livergol, all of which have been characterized in previous phytochemical studies (Table 1). The known biological activities of these compounds are consistent with the reductions in liver enzymes, oxidative stress markers, proinflammatory cytokines (TNF‐α, IL‐1β, and IL‐6), and the histopathological improvements observed in the present MASLD model.

The lack of significant efficacy of flixweed extract in most of the evaluated parameters may be attributed to several mechanistic and methodological factors. Although flixweed has been traditionally used for the management of inflammatory and metabolic disorders, its phytochemical profile differs substantially from those of barberry and jujube. The seeds of flixweed are particularly rich in mucilage polysaccharides, soluble dietary fibers, glucosinolates, and flavonoid glycosides, including quercetin and isorhamnetin derivatives, whereas they contain comparatively lower concentrations of potent alkaloids or highly bioavailable polyphenolic compounds directly targeting hepatic metabolic pathways [30, 69]. Consequently, the beneficial effects of flixweed are believed to occur predominantly through modulation of gastrointestinal physiology and gut microbiota composition rather than through direct hepatocellular actions.

Recent evidence suggests that the polysaccharide‐rich fraction of flixweed can increase SCFA production, improve intestinal barrier integrity, and reduce systemic inflammation through gut–liver axis regulation [69, 70]. However, such mechanisms generally require prolonged administration and may exert relatively modest effects in comparison with compounds such as berberine and silymarin, which directly regulate key hepatic signaling pathways including AMPK, NF‐κB, and Nrf2 [62, 64]. Therefore, the absence of significant changes in inflammatory gene expression and oxidative stress markers observed in the present study may reflect the indirect mode of action of flixweed rather than a complete lack of biological activity.

Higher doses of flixweed may be required to achieve measurable hepatoprotective effects in diet‐induced MASLD. In addition, the hydroalcoholic extraction method used in the present study may not have been optimal for concentrating the high‐molecular‐weight polysaccharides and mucilage fractions that are considered major active constituents of flixweed. Since these compounds are generally more efficiently recovered using aqueous extraction procedures, the phytochemical composition of the administered extract may not have fully reflected the therapeutic potential of the plant [30, 71].

Finally, it should be considered that MASLD is primarily characterized by profound disturbances in hepatic lipid metabolism, oxidative stress, and inflammatory signaling. The known pharmacological profile of flixweed appears to be more closely associated with gastrointestinal regulation and systemic metabolic modulation than with direct inhibition of hepatic lipogenesis or inflammatory cytokine production. This pharmacological distinction may explain why flixweed exhibited only modest and nonsignificant effects in the present model.

### 4.2. Limitations

Limitations of the present study include the following: first, although the MASLD animal model enabled controlled evaluation of the interventions, it does not fully recapitulate the clinical and metabolic complexity of human MASLD, which may limit the direct translation of the findings to clinical settings. Second, the study employed a single dose and fixed treatment duration for each intervention, and comprehensive phytochemical characterization (HPLC) of the hydroalcoholic extracts was not performed. Therefore, dose–response relationships and the contribution of specific bioactive constituents could not be fully determined. Third, although body weight and food intake were monitored throughout the study, liver and adipose tissue weights were not measured at sacrifice, precluding a precise distinction between direct hepatoprotective effects and indirect effects mediated through changes in adiposity. In addition, spontaneous locomotor activity and energy expenditure were not assessed. Therefore, the potential contribution of differences in physical activity to the observed metabolic and hepatic outcomes cannot be completely excluded.

## 5. Conclusion

The findings of this study indicate that hydroalcoholic extracts of barberry, jujube, and livergol can exert significant hepatoprotective effects against MASLD‐induced liver injury through multiple mechanisms, including improvement of lipid profile, reduction of liver enzymes, enhancement of antioxidant defenses, inhibition of proinflammatory gene expression, and upregulation of anti‐inflammatory cytokines. Among these extracts, barberry, due to its berberine content, demonstrated the most comprehensive and potent therapeutic effects and may be considered a promising candidate for future clinical studies in MASLD management. However, conducting controlled clinical trials is essential to confirm the efficacy and safety of these herbal extracts in humans.

## Author Contributions

Yaser Mohammadi contributed to the study conception and design. Yaser Mohammadi, Elham Bahreini, Farzad Sadri, and Taraneh Rezaei performed the animal experiments, sample collection, and biochemical assays. Farzad Sadri and Zahra Hemmati carried out the molecular analyses, including RNA extraction, cDNA synthesis, and RT‐qPCR for inflammatory gene expression. Mohammad Babaei performed histopathology of the liver tissue. Elham Bahreini and Yaser Mohammadi supervised the overall project, secured funding, and provided critical intellectual input. Yaser Mohammadi, Farzad Sadri, and Taraneh Rezaei analyzed and interpreted the data. The first draft of the manuscript was prepared by Yaser Mohammadi, and all authors critically revised the manuscript for important intellectual content. The authors take full responsibility for the accuracy and integrity of the publication.

## Funding

This research was supported by the Iran University of Medical Sciences (Grant 31180).

## Disclosure

All authors have read and approved the final version of the manuscript. After using this ChatGPT and OpenAI, the authors carefully reviewed, verified, and edited all content as necessary.

## Ethics Statement

This study was approved by the Ethics Committee of the Iran University of Medical Sciences (Ethics Code IR.IUMS.AEC.1403.068). All animal procedures were performed in compliance with the Guide for the Care and Use of Laboratory Animals and were approved by the Institutional Animal Care and Use Committee of the Iran University of Medical Sciences.

## Consent

The authors have nothing to report.

## Conflicts of Interest

The authors declare no conflicts of interest.

## Data Availability

The present study data are available from the corresponding author upon reasonable request.
